# The CXC Chemokine Receptor 3 Inhibits Autoimmune Cholangitis *via* CD8^+^ T Cells but Promotes Colitis *via* CD4^+^ T Cells

**DOI:** 10.3389/fimmu.2018.01090

**Published:** 2018-05-17

**Authors:** Qing-Zhi Liu, Wen-Tao Ma, Jing-Bo Yang, Zhi-Bin Zhao, Kai Yan, Yuan Yao, Liang Li, Qi Miao, M. Eric Gershwin, Zhe-Xiong Lian

**Affiliations:** ^1^Liver Immunology Laboratory, School of Life Sciences, University of Science and Technology of China, Hefei, China; ^2^Chronic Disease Laboratory, School of Medicine, Institutes for Life Sciences, South China University of Technology, Guangzhou, China; ^3^College of Veterinary Medicine, Northwest Agriculture and Forestry University, Yangling, China; ^4^Department of Gastroenterology and Hepatology, Renji Hospital, School of Medicine, Shanghai Jiao Tong University, Shanghai, China; ^5^Division of Rheumatology, Allergy and Clinical Immunology, School of Medicine, University of California, Davis, Davis, CA, United States

**Keywords:** autoimmune cholangitis, CD4^+^ T cells, CD8^+^ T cells, colitis, CXC chemokine receptor 3, interferon-γ, interleukin-17A, PD-1

## Abstract

CXC chemokine receptor 3 (CXCR3), a receptor for the C-X-C motif chemokines (CXCL) CXCL9, CXCL10, and CXCL11, which not only plays a role in chemotaxis but also regulates differentiation and development of memory and effector T cell populations. Herein, we explored the function of CXCR3 in the modulation of different organ-specific autoimmune diseases in interleukin (IL)-2 receptor deficiency (CD25^−/−^) mice, a murine model for both cholangitis and colitis. We observed higher levels of CXCL9 and CXCL10 in the liver and colon and higher expression of CXCR3 on T cells of the CD25^−/−^ mice compared with control animals. Deletion of CXCR3 resulted in enhanced liver inflammation but alleviated colitis. These changes in liver and colon pathology after CXCR3 deletion were associated with increased numbers of hepatic CD4^+^ and CD8^+^ T cells, in particular effector memory CD8^+^ T cells, as well as decreased T cells in mesenteric lymph nodes and colon lamina propria. In addition, increased interferon-γ response and decreased IL-17A response was observed in both liver and colon after CXCR3 deletion. CXCR3 modulated the functions of T cells involved in different autoimmune diseases, whereas the consequence of such modulation was organ-specific regarding to their effects on disease severity. Our findings emphasize the importance of extra caution in immunotherapy for organ-specific autoimmune diseases, as therapeutic interventions aiming at a target such as CXCR3 for certain disease could result in adverse effects in an unrelated organ.

## Introduction

CXC chemokine receptor 3 (CXCR3) is considered as a type 1 T help cell (Th1) receptor highly expressed on activated T, natural killer (NK), and B cells. There are three interferon (IFN)-γ-induced ligands for CXCR3, the C-X-C motif chemokine (CXCL) 9, CXCL10, and CXCL11 (1, 2). Together with its ligands, CXCR3 plays roles in many diseases (3). Previous works have focused on the chemotaxis function of CXCR3 in autoimmune diseases and cancer. CXCR3 blockage usually results in attenuation of inflammation due to decreased infiltration of autoreactive CD4^+^ T cells and CD8^+^ T cells in autoimmune diseases (4–7). CXCR3 expression on tumor cells promotes cancer metastasis, while its expression on T cells regulates their antitumor reactivity (8, 9). In particular, the role of CXCR3 in different autoimmune diseases has not been fully elucidated, due to the complexity of its functions and different effects of CXCR3 on various diseases (10–12).

Primary biliary cholangitis (PBC) is an autoimmune liver disease characterized by specific destruction of small bile ducts (13). In PBC patients, CXCR3 is expressed at high levels on PBMC while its ligands are also present at high levels in the plasma (14). Demethylation in the promoter of CXCR3 gene in CD4^+^ T cells has been detected in PBC patients (15). Several studies have reported a pathogenic role of CXCR3 in autoimmune colitis (16–18). In our previous work with a mouse model for both PBC and colitis, the interleukin (IL)-2 receptor deficiency (CD25^−/−^) mice, we revealed different mechanisms in the pathogenesis of cholangitis versus colitis (19, 20). In the current study, we took advantage of this mice to explore the roles of CXCR3 in the autoimmune diseases of different organs by examining the effects of deleting this gene on the infiltrating T cells and disease severity in the liver and colon. Our findings provide new insights to the CXCR3-related mechanisms for organ-specific autoimmune diseases.

## Materials and Methods

### Mice

CD25^−/−^ (B6.129S4-Il2ra^tm1Dw/J^) mice were purchased from Jackson Laboratory. CD25^+/−^ and CD25^−/−^ mice used in the experiments were littermates from heterozygote propagation of CD25^+/−^ mice. CXCR3^−/−^ mice were kindly provided by Dr. Bao Lu (Harvard Medical School, MA, USA). The expression level of CXCR3 on T cells was determined in male and female CD25^+/−^ and CD25^−/−^ mice at the age of 11–13 weeks by flow cytometry. The samples for RNA and protein levels of CXCL9/CXCL10 detection were from different groups of mice. 10- to 13-week-old CXCR3^+/−^ mice and CXCR3^−/−^ mice regardless of gender were used to measure liver and colon inflammation. CD25^−/−^CXCR3^−/−^ mice were generated by crossing CD25^+/−^ mice with CXCR3^−/−^ mice. The roles of CXCR3 in autoimmune cholangitis and colitis were examined in 10- to 13-week-old CD25^−/−^ and CD25^−/−^CXCR3^−/−^ mice regardless of gender. The effects of CXCR3 on autoimmune cholangitis were measured by histopathology and hepatic mononuclear cell (MNC) counts. The effects of CXCR3 on colitis were measured by histopathology, colon weight, and mesenteric lymph node (MLN) MNC counts. T cell homeostasis was studied in the same mice by flow cytometric analyses of cell surface markers (CD44, CD62L, KLRG1, and PD-1), transcription factor T-bet, and intracellular cytokines (IFN-γ and IL-17A) compared CD25^−/−^ with CD25^−/−^CXCR3^−/−^ mice. The transcription factors RORγt and Ki67 were detected in different mice groups. Another group of 10- to 13-week-old CD25^−/−^ and CD25^−/−^CXCR3^−/−^ mice were used for detecting the relationship between PD-1 and intracellular cytokines (IFN-γ and IL-17A) in liver and MLN. All results about the colon lamina propria lymphocytes (LPL) were from another group of 10- to 13-week-old CD25^−/−^ and CD25^−/−^CXCR3^−/−^ mice. CCR4, CCR5, CCR6, and IL-21 and its receptor IL-21R were detected in 15-week-old CD25^−/−^ and CD25^−/−^CXCR3^−/−^ mice regardless of gender. All mice used in the experiments were of B6 background and bred under special pathogen-free conditions (22°C, 55% humidity, and 12-h day/night rhythm) according to the Guidelines for the Care and Use of Laboratory Animals of the University of Science and Technology of China (Hefei, Anhui, China).

### Histopathology

Liver and colon tissues were fixed in 4% paraformaldehyde for 48 h, then cut into 5 µm sections for H&E staining. The histology scores for portal inflammation, bile duct damage, and colitis were determined as previously described (20).

### Flow Cytometry

Mononuclear cells from liver, spleen, and MLN were isolated and stained as described (10). Colon LPL were isolated using a standardized protocol (21). Collagenase IV (Sigma-Aldrich, St. Louis, MO, USA) was used at a final concentration of 0.5 mg/ml. For intracellular cytokine staining, MNC from liver, MLN, and LPL were first stimulated with cell stimulation cocktail (eBioscience, San Diego, CA, USA) in RPMI 1640 medium (Thermo Fisher Scientific, USA) supplemented with 10% fetal bovine serum (Millipore, Billerica, MA, USA) at 37°C for 4 h. The cells were fixed and permeabilized with BD Cytofix/Cytoperm™ Fixation/Permeabilization Kit (BD Bioscience, San Diego, CA, USA) according to the manufacturer’s instructions. For transcription factor and nuclear Ki67 protein staining, Foxp3 staining buffer set (eBioscience, San Diego, CA, USA) was used according to the manufacturer’s instructions. PB-conjugated anti-CD3 (17A2), FITC-conjugated anti-CD3 (17A2) and anti-CD44 (IM7), PerCP/Cy5.5-conjugated anti-CD4 (GK1.5), anti-CD62L (MEL-14) and anti-T-bet (4B10), PE-conjugated antibodies against CXCR3 (CXCR3-173), PD-1 (RMP1-30), IFN-γ (XMG1.2), IL-17A (TC11-18H10.1), CCR6 (29-2L17), CCR4 (2G12), Ki67 (16A8), and CD62L (MEL-14), PE/Cy7-conjugated anti-NK1.1 (PK136) and anti-IFN-γ (XMG1.2), APC-conjugated antibodies against PD-1 (29F.1A12), KLRG1 (2F1/KLRG-1), CD44 (IM7), CCR5 (HM-CCR5), and APC/Cy7-conjugated anti-CD8a (53-6.7) were purchased from BioLegend (San Diego, CA, USA). V500-conjugated anti-CD4 (RM4-5) was purchased from BD Biosciences. APC-conjugated antibodies against RORγt (AFKJS-9) were purchased from eBiosciences.

Flow cytometric data were acquired using a FACSVerse flow cytometer (BD Biosciences) and analyzed with the Flowjo software (Tree Star, Ashland, OR, USA).

### Enzyme-Linked Immunosorbent Assays (ELISA)

Liver tissues were weighted and homogenized with 1× PBS, then stored at −80°C. The homogenized tissues were repeatedly frozen and thawed, then centrifuged at 6,000 *g* for 3 min to collect supernatants. Colon tissues were weighted, cut, and homogenized in PBS with an ultrasonic disruptor (Xinzhi, Ningbo, China), then centrifuged at 6,000 *g* for 3 min to collect supernatants. The levels of CXCL9 and CXCL10 in serum, liver, and colon homogenate supernatants were measured using ELISA kits from Cusabio (Wuhan, China) according to the manufacturer’s instructions.

### Magnetic-Activated Cell Sorting

CD4^+^ T and CD8^+^ T cells were separately sorted from CD25^−/−^ and CD25^−/−^CXCR3^−/−^ mice using mouse CD4 (L3T4) and CD8a (Ly-2) MicroBeads (Miltenyi Biotec, Germany) according to the manufacturer’s instructions. CD4^+^ T and CD8^+^ T cells purity was more than 95%.

### RNA Preparation, Reverse Transcription, and Quantitative Real-Time PCR

Total RNA was extracted from the liver and colon tissues of CD25^+/−^ and CD25^−/−^ mice using RNAiso Plus (Takara, Kusatsu, Shiga, Japan). Total RNA was extracted from sorted CD4^+^ T and CD8^+^ T cells by using Trizol (Invitrogen, Carlsbad, CA, USA). PrimeScript^RT^ Reagent Kit with gDNA Eraser (Takara, Kusatsu, Shiga, Japan) was used for reverse transcription and quantitative real-time PCR according to the manufacturer’s instructions. Results for all target genes were normalized to that of the housekeeping gene GAPDH and used for calculating 2^−ΔΔCt^ (22). The PCR primers used were GAPDH, 5′-AACTTTGGCATTGTGGAAGG-3′ and 5′-ACACATTGGGGGTAGGAACA-3′; CXCL9, 5′-AATGCACGATGCTCCTGCA-3′ and 5′-AGGTCTTTGAGGGATTTGTAGTGG-3′; and CXCL10, 5′-GCCGTCATTTTCTGCCTCA-3′ and 5′-CGTCCTTGCGAGAGGGATC-3′. CCR4, 5′-GGAAGGTATCAAGGCATTTGGG-3′ and 5′-GTACACGTCCGTCATGGACTT-3′; CCR5, 5′-TTTTCAAGGGTCAGTTCCGAC-3′ and 5′-GGAAGACCATCATGTTACCCAC-3′; CCR6, 5′-GCTCCAGAACACTGACGCA-3′ and 5′-CTGTACCGTGGCTCACAGA-3′; IL-21, 5′-CTTCGTCACCTTATTGACATTGTTG-3′ and 5′-CCAGGGTTTGATGGCTTGA-3′; and IL-21R, 5′-GGCTGCCTTACTCCTGCTG-3′ and 5′-TCATCTTGCCAGGTGAGACTG-3′. All primers were synthesized by Sangon Biotech (Shanghai, China).

### Statistical Analysis

All data were presented as mean ± SD. All data were analyzed by SPSS software for Kolmogorov–Smirnov test and all test distributions were normal (*p* > 0.05). The comparison between groups was performed with two-tailed unpaired Student’s *t* test. *p* Value of <0.05 was considered as statistically significant.

## Results

### Increased Expression of CXCR3 and Its Ligands in Liver and Colon of CD25^−/−^ Mice

**Figure 1:**
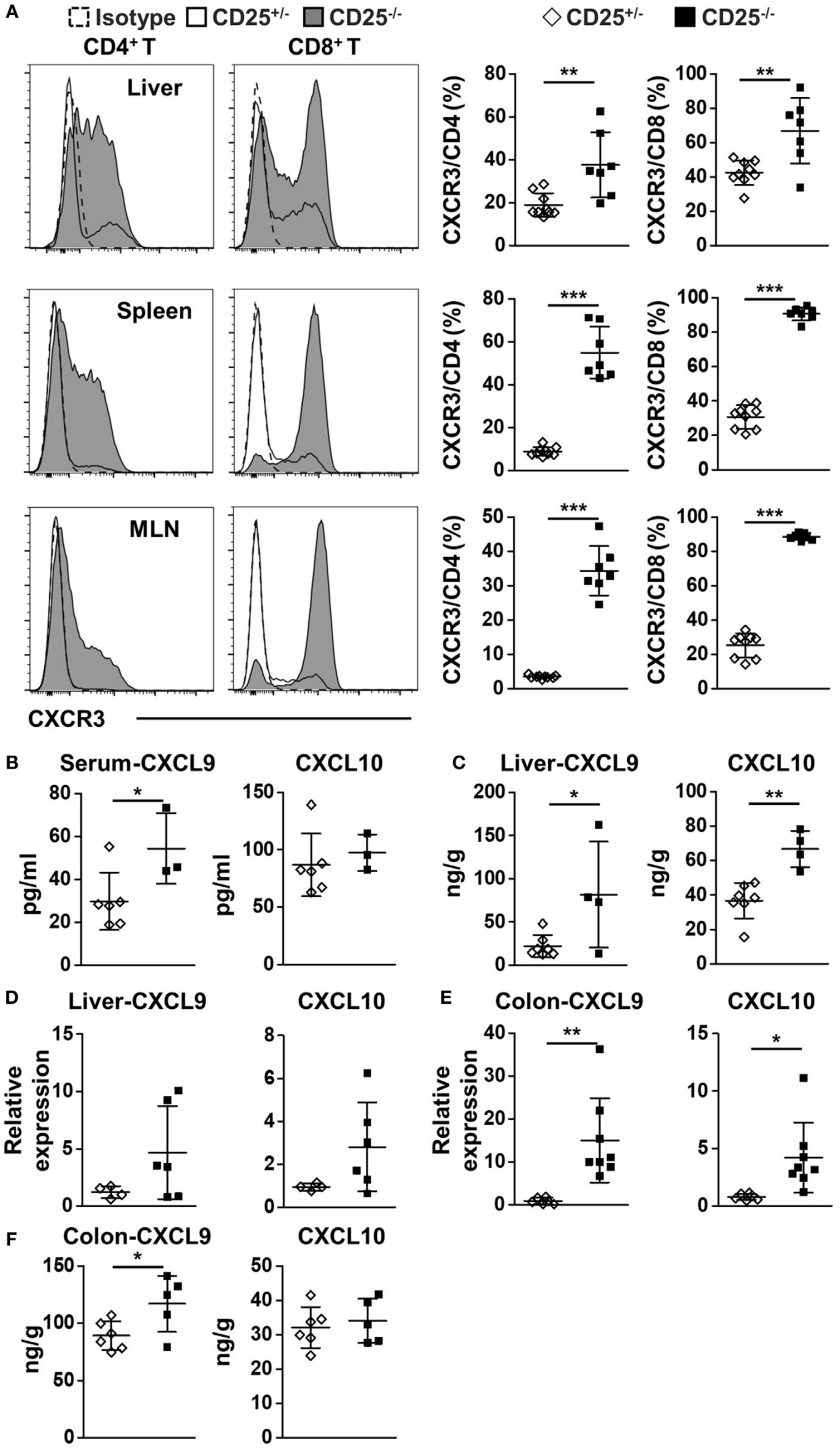
Increased expression of CXC chemokine receptor 3 (CXCR3) and its ligands in liver and colon of CD25^−/−^ mice in comparison to their CD25^+/−^ littermates. **(A)** The percentages of CXCR3^+^CD4^+^ and CXCR3^+^CD8^+^ T cells in liver, spleen, and mesenteric lymph nodes (MLN) were analyzed by flow cytometry and compared between the CD25^+/−^ mice (*N* = 9) and CD25^−/−^ mice (*N* = 7). **(B)** The concentration of CXCL9 and CXCL10 in serum was measured by enzyme-linked immunosorbent assays (ELISA) and compared between CD25^+/−^ mice (*N* = 6) and CD25^−/−^ mice (*N* = 3). **(C)** The concentration of CXCL9 and CXCL10 in liver homogenate was measured by ELISA and compared between CD25^+/−^ mice (*N* = 7) and CD25^−/−^ mice (*N* = 4). The relative mRNA levels of CXCL9 and CXCL10 in liver **(D)** and colon **(E)** were measured by quantitative real-time PCR and compared between CD25^+/−^ mice (*N* = 6) and CD25^−/−^ mice (*N* = 6–8). **(F)** The concentration of CXCL9 and CXCL10 in colon homogenate was measured by ELISA and compared between CD25^+/−^ mice (*N* = 6) and CD25^−/−^ mice (*N* = 5). *p < 0.05; **p < 0.01; ***p < 0.001.

We first examined the expression of CXCR3 in the CD4^+^ and CD8^+^ T cell populations in the CD25^−/−^ mice in comparison to their CD25^+/−^ littermates. The percentages of CXCR3^+^ cells in CD4^+^ T cells from the liver, spleen, and MLN were all significantly higher in CD25^−/−^ mice than CD25^+/−^ littermates (*p* = 0.0039 for liver, *p* < 0.0001 for spleen, and *p* < 0.0001 for MLN) (Figure 1A). The percentages of CXCR3^+^ cells in CD8^+^ T cells of CD25^−/−^ mice also increased in liver, spleen, and MLN (*p* = 0.0032 for liver, *p* < 0.0001 for spleen, and *p* < 0.0001 for MLN) (Figure 1A). Next, we examined the levels of the two CXCR3 ligands, CXCL9 and CXCL10. Serum level of CXCL9 was significantly increased (*p* = 0.0450) in CD25^−/−^ mice in comparison to their CD25^+/−^ littermates, whereas significant difference was not detected in the serum level of CXCL10 (Figure 1B). Importantly, protein levels of CXCL9 (*p* = 0.0295) and CXCL10 (*p* = 0.0012) were both significantly higher in liver homogenate of CD25^−/−^ mice than that of their CD25^+/−^ littermates (Figure 1C). The average mRNA levels of these chemokine genes in the liver were also higher in the CD25^−/−^ mice although the difference did not reach statistical significance (Figure 1D). mRNA levels of CXCL9 (*p* = 0.0045) and CXCL10 (*p* = 0.0196) in colon were significantly higher in the CD25^−/−^ mice (Figure 1E). The average protein levels of both CXCL9 and CXCL10 (*p* = 0.6183) in colon homogenate were also higher in the CD25^−/−^ mice, although the difference reached statistical significance for CXCL9 only (*p* = 0.0377) (Figure 1F). Taken together, these results indicate that in this mouse model for autoimmune cholangitis and colitis, expression of CXCR3 and its ligands are increased in the liver and colon.

### Different Expression Patterns of CXCR3 in T Cell Subsets

**Figure 2:**
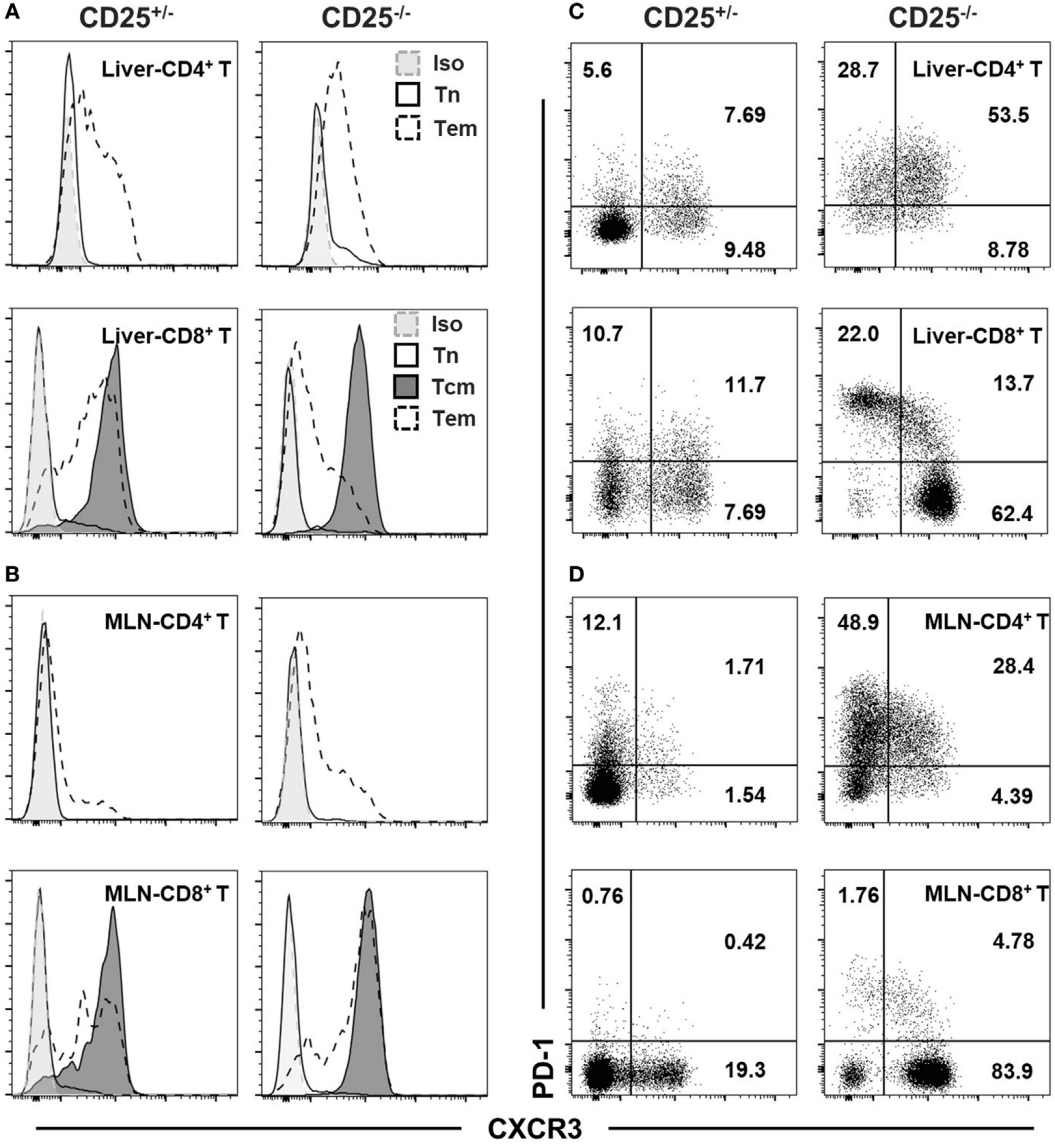
CXC chemokine receptor 3 (CXCR3) and PD-1 phenotypes of T cell subsets in CD25^−/−^ mice and their CD25^+/−^ littermates. Expression of CXCR3 on naïve T (Tn, CD62L^+^CD44^−^), central memory T (Tcm, CD62L^+^CD44^+^), and effect memory T (Tem, CD62L^−^CD44^+^) cells among hepatic **(A)** and mesenteric lymph node (MLN) **(B)** CD4^+^ T and CD8^+^ T cells were examined by flow cytometry. CD4^+^ Tcm were not examined due to their low frequency. The expression patterns of CXCR3 and PD-1 in hepatic **(C)** and MLN **(D)** CD4^+^ T and CD8^+^ T cells were also compared between CD25^+/−^ and CD25^−/−^ mice. At least five mice in each group were examined and generated data similar to the representative results shown in this figure.

We further examined the expression level of CXCR3 in different T cell subsets. The expression levels of CXCR3 differed between naïve (Tn: CD62L^+^CD44^−^), central memory (Tcm: CD62L^+^CD44^+^), and effector memory (Tem: CD62L^−^CD44^+^) T cell subsets in liver (Figure 2A) and MLN (Figure 2B) of both CD25^+/−^ and CD25^−/−^ mice. For both CD4^+^ T and CD8^+^ T cells from liver and MLN in CD25^−/−^ mice and their CD25^+/−^ littermates, naïve cells expressed minimum level of CXCR3. Tcm cells in the CD8^+^ T cell population were all CXCR3^+^, whereas only a part of Tem cells in both CD25^+/−^ and CD25^−/−^ mice expressed CXCR3 (Figures 2A,B). Finally, we examined the relationship between expression levels of CXCR3 and the T cell exhaustion marker PD-1. In CD25^−/−^ mice, PD-1 was co-expressed with CXCR3 on approximately 50% of hepatic CD4^+^ T cells. By contrast, only 10% of CD8^+^ T cells were PD-1^+^CXCR3^+^ and the majority of PD-1^hi^CD8^+^ T cells did not express CXCR3 in the liver (Figure 2C). Strikingly different from the liver, only approximately 30% of MLN CD4^+^ T cells were PD-1^+^CXCR3^+^ and approximately 50% of CD4^+^ T cells were CXCR3^−^. For MLN CD8^+^ T cells, less than 10% expressed PD-1 and the majority of the cells were PD-1^−^CXCR3^+^ in the CD25^−/−^ mice (Figure 2D). Besides, approximately 90% of PD-1^+^ T cells were Tem both in liver and MLN of CD25^−/−^ mice (Figure S1 in Supplementary Material). These results show that CXCR3 not only is differently expressed on Tcm and Tem cells but also has different expression patterns with PD-1 on CD4^+^ T and CD8^+^ T cells from liver and colon, respectively, in the CD25^−/−^ mouse model.

### CXCR3 Deletion Aggravates Liver Inflammation of CD25^−/−^ Mice

**Figure 3:**
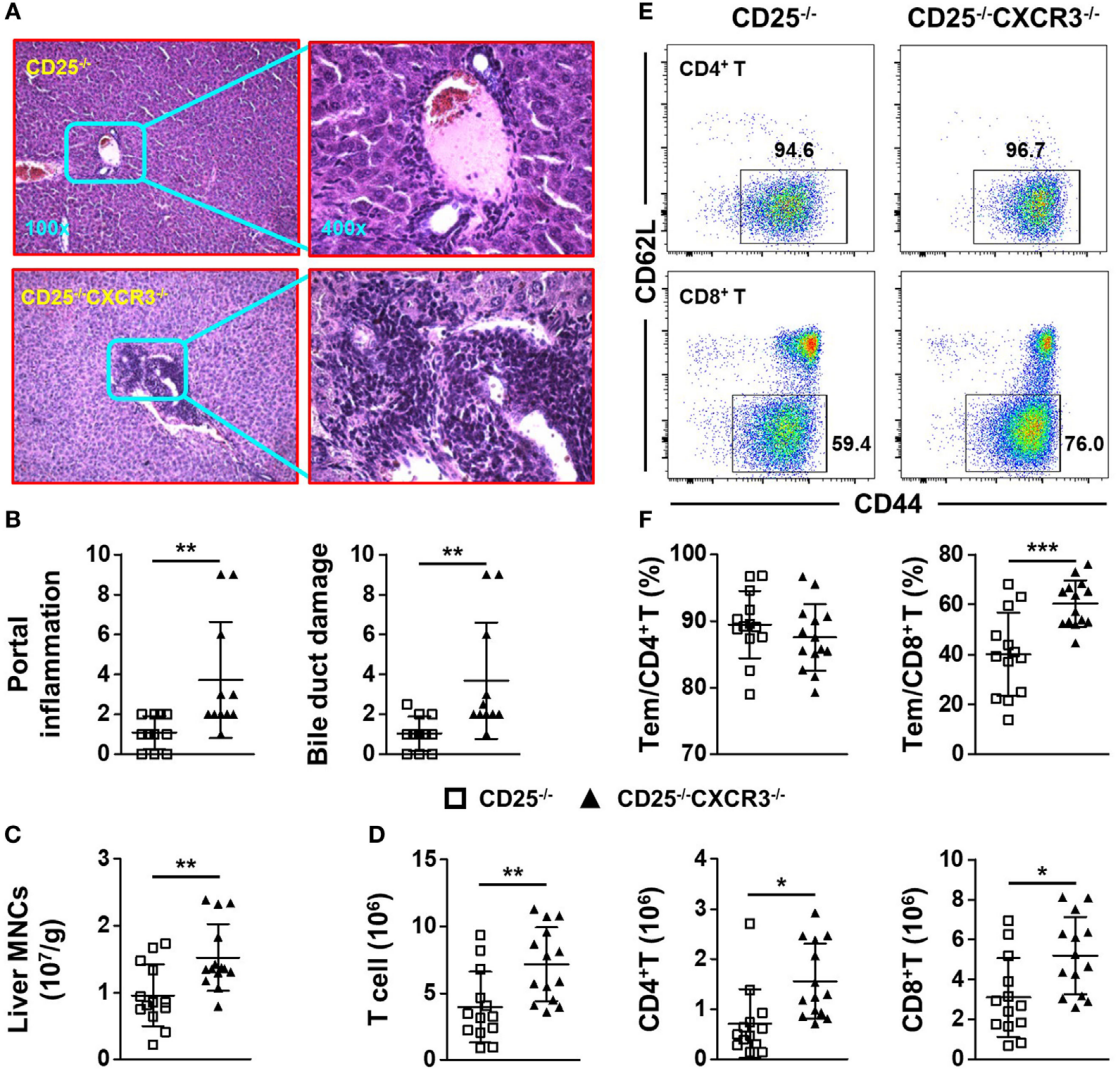
CXC chemokine receptor 3 (CXCR3) deletion aggravates liver inflammation of CD25^−/−^ mice. **(A)** Representative H&E-stained liver sections of CD25^−/−^ and CD25^−/−^CXCR3^−/−^ mice at the original magnification of 100× and 400×. **(B)** Scores of portal inflammation and bile duct damage in CD25^−/−^ mice (*N* = 11) and CD25^−/−^CXCR3^−/−^ mice (*N* = 11). **(C)** Numbers of liver mononuclear cells (MNCs) per gram of liver weight in CD25^−/−^ mice (*N* = 13) and CD25^−/−^ CXCR3^−/−^ mice (*N* = 14). **(D)** Numbers of liver T, CD4^+^ T, and CD8^+^ T cells from CD25^−/−^ mice (*N* = 13) and CD25^−/−^CXCR3^−/−^ mice (*N* = 14). **(E)** Representative flow cytometry dot plots of CD44 and CD62L expression on CD4^+^ T and CD8^+^ T cells from CD25^−/−^ mice and CD25^−/−^CXCR3^−/−^ mice. **(F)** Percentages of effector memory (Tem) cells in hepatic CD4^+^ T and CD8^+^ T populations of CD25^−/−^ mice (*N* = 13) and CD25^−/−^ CXCR3^−/−^ mice (*N* = 14). *p < 0.05; **p < 0.01; ***p < 0.001.

To explore the role of CXCR3 in cholangitis and colitis, we crossed CD25^+/−^ mice with CXCR3^−/−^ mice to generate CD25^−/−^CXCR3^−/−^ mice. First, we detected the effects of CXCR3 on liver and colon inflammation in CXCR3^+/−^ and CXCR3^−/−^ mice and found no difference between the two groups (Figure S2 in Supplementary Material). Next based on H&E staining and pathology scores, portal inflammation (*p* = 0.0089) and bile duct damage (*p* = 0.0093) were aggravated after CXCR3 deletion (Figures 3A,B). Consistent with exacerbated liver inflammation, the MNC number per gram of liver weight was also increased (*p* = 0.0052) (Figure 3C). In addition, the absolute numbers of T cells (*p* = 0.0052), CD4^+^ T cells (*p* = 0.0052), and CD8^+^ T cells (*p* = 0.0100) in liver were all increased in the CD25^−/−^CXCR3^−/−^ mice (Figure 3D). By contrast, the total number of splenocytes did not differ significantly between the CD25^−/−^ and CD25^−/−^CXCR3^−/−^ mice (Figure S5A in Supplementary Material). The increased T cells were not due to cell proliferation or increased expression of other chemokine receptors (Figures S3A,B and S4A–D in Supplementary Material). We also examined the effects of CXCR3 deletion on hepatic T cell subsets. After CXCR3 deletion, the percentage of CD8^+^ Tem, but not CD4^+^ Tem, significantly increased (*p* = 0.0005 for CD8^+^ Tem, *p* = 0.3277 for CD4^+^ Tem) (Figures 3E,F). These results suggest that CXCR3 has a protective role against the autoimmune cholangitis in the CD25^−/−^ mouse model.

### CXCR3 Deletion Alleviates Colitis of CD25^−/−^ Mice

**Figure 4:**
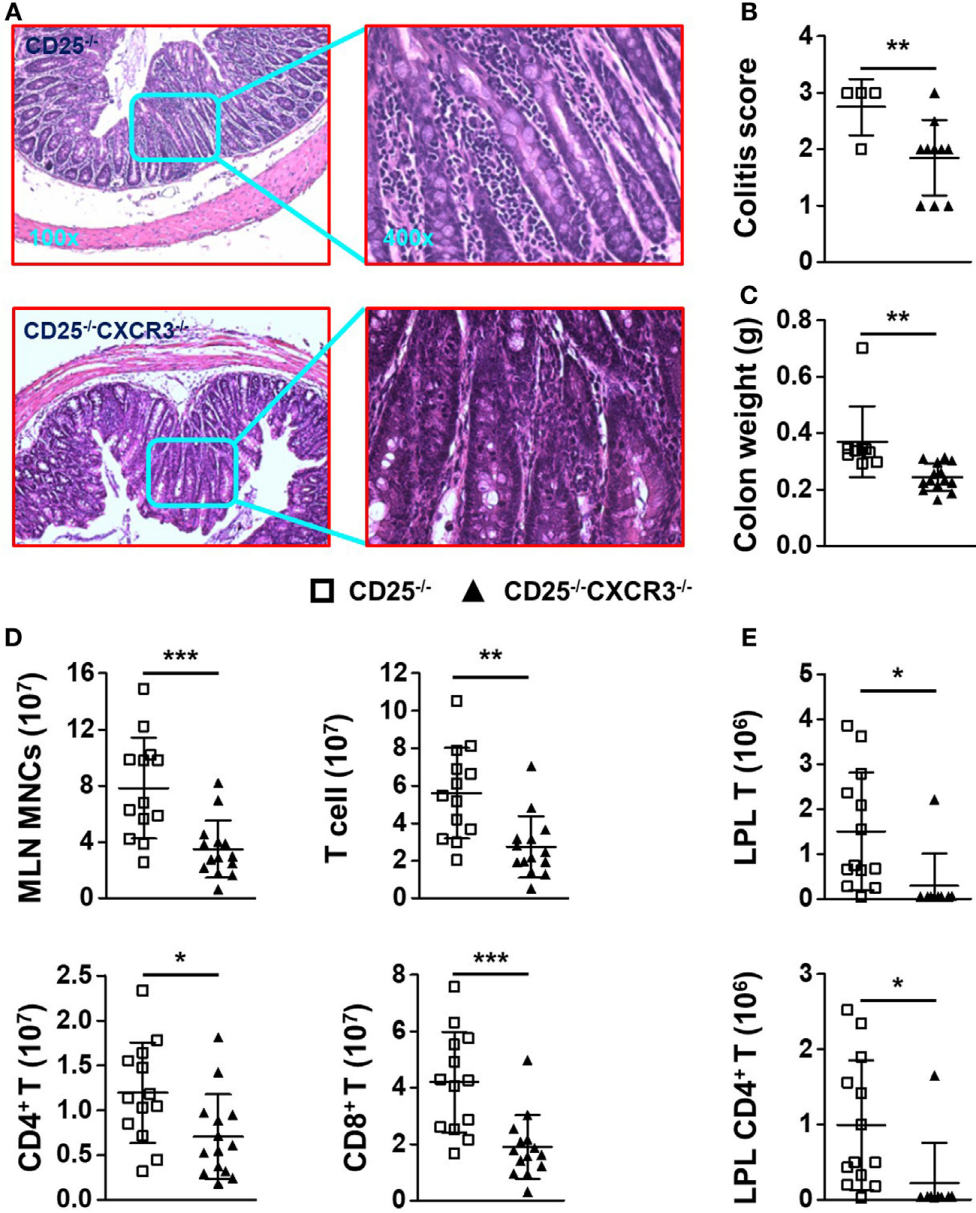
CXC chemokine receptor 3 (CXCR3) deletion alleviates colitis of CD25^−/−^ mice. **(A)** Representative H&E-stained colon sections of CD25^−/−^ and CD25^−/−^CXCR3^−/−^ mice at the original magnification of 100× and 400×. **(B)** Scores of colitis in CD25^−/−^ mice (*N* = 4) and CD25^−/−^CXCR3^−/−^ mice (*N* = 10). **(C)** Colon weight of CD25^−/−^ mice (*N* = 7) and CD25^−/−^CXCR3^−/−^ mice (*N* = 10). **(D)** Numbers of mononuclear cell (MNC), T, CD4^+^ T, and CD8^+^ T cells in mesenteric lymph nodes (MLN) from CD25^−/−^ mice (*N* = 13) and CD25^−/−^CXCR3^−/−^ mice (*N* = 14). **(E)** Numbers of T and CD4^+^ T cells in colon lamina propria lymphocytes (LPL) from CD25^−/−^ mice (*N* = 13) and CD25^−/−^CXCR3^−/−^ mice (*N* = 9). *p < 0.05; **p < 0.01; ***p < 0.001.

Surprisingly, the colitis of CD25^−/−^ mice was alleviated after CXCR3 deletion, which was indicated by H&E staining of colon (Figure 4A), score of colitis (*p* = 0.0328) (Figure 4B), and colon weight (*p* = 0.0027) (Figure 4C). The absolute number of MLN MNC decreased significantly when CXCR3 was deleted in the CD25^−/−^ mice (*p* = 0.0006) (Figure 4D). In addition, the numbers of total MLN T, CD4^+^ T, and CD8^+^ T cells all decreased (*p* = 0.0012 for T, *p* = 0.0216 for CD4^+^ T, and *p* = 0.0004 for CD8^+^ T) (Figure 4D). The relative mean fluorescent intensity (RMFI) of Ki67 was decreased both in MLN CD4^+^ T (*p* = 0.0003) and CD8^+^ T cells (*p* = 0.0383) of CD25^−/−^CXCR3^−/−^ mice (Figures S3A,B in Supplementary Material). Though the RNA level of chemokine receptors CCR6 (*p* = 0.0182) on MLN CD4^+^ T cells was decreased, RNA levels of CCR4 (*p* = 0.0030) and CCR5 (*p* = 0.0014) were increased in CD25^−/−^CXCR3^−/−^ mice compared to CD25^−/−^ mice. However, the protein levels of CCR4, CCR5, and CCR6 were same between CD25^−/−^ and CD25^−/−^CXCR3^−/−^ mice (Figures S4E,F in Supplementary Material). The absolute number of T, CD4^+^ T, and CD8^+^ T cells also decreased in colonic LPL of the CD25^−/−^CXCR3^−/−^ mice (*p* = 0.0208 for T, *p* = 0.0275 for CD4^+^ T, and *p* = 0.0125 for CD8^+^ T) (Figure 4E; Figure S5E in Supplementary Material). These results suggest that CXCR3 is pathogenic for the autoimmune colitis in the CD25^−/−^ mouse model.

### CXCR3 Deletion Elevates Levels of Pro-Inflammatory Factors in the Liver of CD25^−/−^ Mice

**Figure 5:**
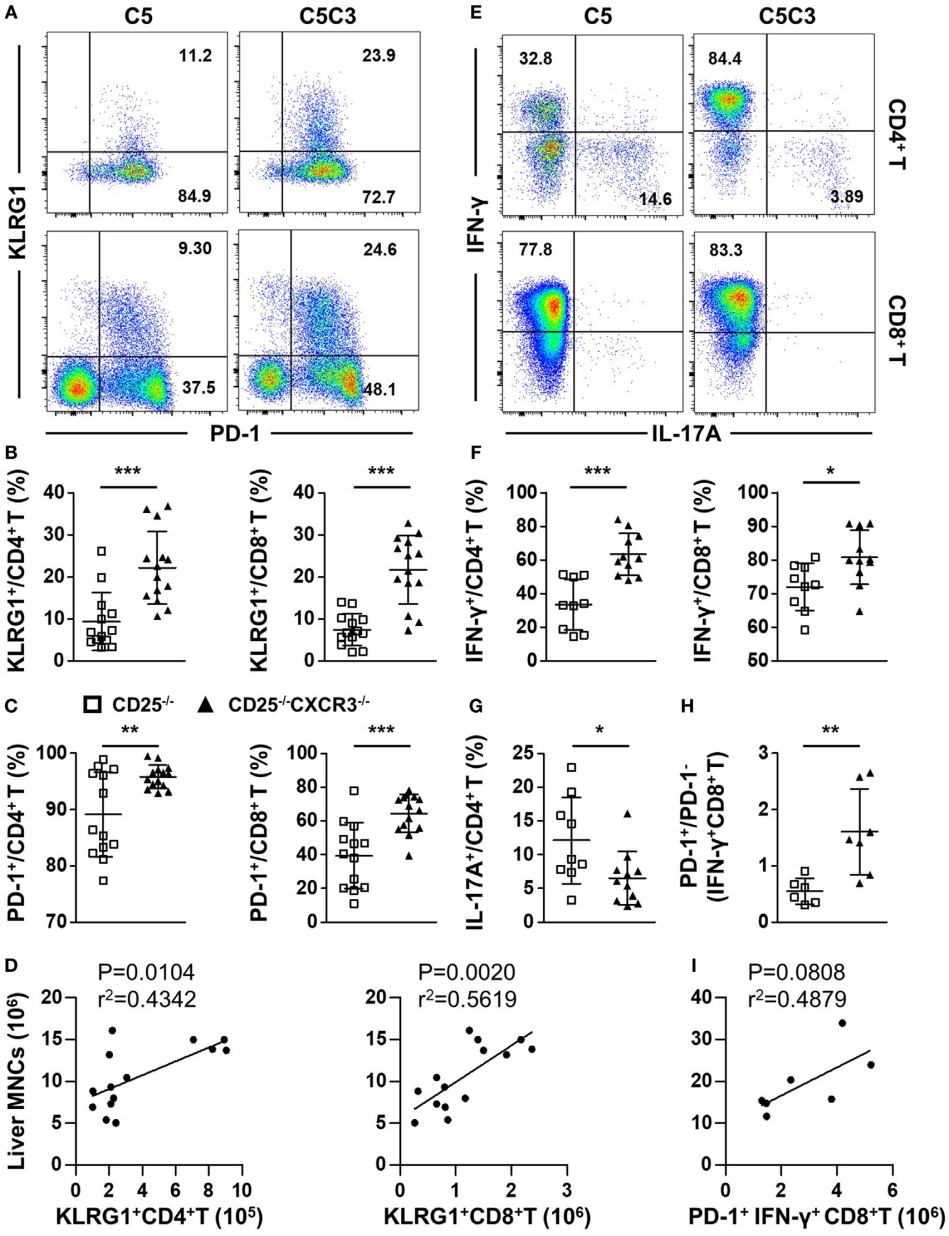
Effects of CXC chemokine receptor 3 (CXCR3) deletion on T cell exhaustion markers and pro-inflammatory factors in liver of CD25^−/−^ mice. The phenotypes of T cell subsets in the CD25^−/−^ and CD25^−/−^CXCR3^−/−^ mice were analyzed with flow cytometry. **(A)** Representative flow cytometry dot plots of hepatic CD4^+^ and CD8^+^ T cells stained for PD-1 and KLRG1. **(B)** Percentage of KLRG1^+^ cells in hepatic CD4^+^ T and CD8^+^ T populations of CD25^−/−^ mice (*N* = 13) and CD25^−/−^CXCR3^−/−^ mice (*N* = 14). **(C)** Percentage of CD-1^+^ cells in hepatic CD4^+^ T and CD8^+^ T populations of CD25^−/−^ mice (*N* = 13) and CD25^−/−^CXCR3^−/−^ mice (*N* = 14). **(D)** The correlation analysis between liver mononuclear cells (MNCs) and hepatic KLRG1^+^CD4^+^ T and KLRG1^+^CD8^+^ T cells from CD25^−/−^CXCR3^−/−^ mice (*N* = 14). **(E)** Representative flow cytometry dot plots of hepatic CD4^+^ and CD8^+^ T cells stained for intracellular cytokines interferon (IFN)-γ and interleukin (IL)-17A. **(F)** Percentages of IFN-γ-producing CD4^+^ T and CD8^+^ T cells in liver of CD25^−/−^ mice (*N* = 9) and CD25^−/−^ CXCR3^−/−^ mice (*N* = 11). **(G)** Percentage of IL-17A-producing CD4^+^ T cells in liver of CD25^−/−^ mice (*N* = 9) and CD25^−/−^ CXCR3^−/−^ mice (*N* = 11). **(H)** PD-1^+^/PD-1^−^ ratio in IFN-γ-producing hepatic CD8^+^ T cells compared between CD25^−/−^ mice (*N* = 6) and CD25^−/−^ CXCR3^−/−^ mice (*N* = 7). **(I)** The correlation analysis between liver MNCs and hepatic PD-1^+^IFN-γ^+^CD4^+^ T cells from CD25^−/−^CXCR3^−/−^ mice (*N* = 7). *p < 0.05; **p < 0.01; ***p < 0.001. C5, CD25^−/−^ mice; C5C3, CD25^−/−^CXCR3^−/−^ mice.

We first compared the expression of T cell exhaustion markers PD-1 and KLRG1 on hepatic CD4^+^ and CD8^+^ T cells from CD25^−/−^ and CD25^−/−^CXCR3^−/−^ mice. The percentages of CD4^+^ and CD8^+^ T cells that express PD-1 and KLRG1 significantly increased in the liver of CD25^−/−^CXCR3^−/−^ mice compared to CD25^−/−^ mice (*p* = 0.0002 for KLRG1^+^ CD4^+^ T, *p* < 0.0001 for KLRG1^+^ CD8^+^ T, *p* = 0.0037 for PD-1^+^ CD4^+^ T, and *p* = 0.0003 for PD-1^+^ CD8^+^ T) (Figures 5A–C). The absolute number of hepatic KLRG1^+^ T cells positively correlated with liver MNC of CD25^−/−^CXCR3^−/−^ mice (Figure 5D). Next, we examined the cytokine secretion ability of hepatic CD4^+^ and CD8^+^ T cells. IFN-γ secreting ability of liver CD4^+^ and CD8^+^ T cells increased (*p* = 0.0001 for CD4^+^ T cells and *p* = 0.0190 for CD8^+^ T cells) (Figures 5E,F) but IL-17A secreting ability decreased (*p* = 0.0272 for CD4^+^ T cells) (Figures 5E,G) with deletion of CXCR3. But IFN-γ secreting ability of splenic T cells increased only in CD4^+^ not CD8^+^ T cells (*p* < 0.0001 for CD4^+^ T cells, Figure S5A in Supplementary Material). And the RNA levels of transcription factors *T-bet* and *RORγt* on hepatic CD4^+^ T cells were same between CD25^−/−^ and CD25^−/−^CXCR3^−/−^ mice (Figures S3E,F in Supplementary Material). We also detected the RNA levels of cytokine IL-21 on CD4^+^ T and cytokine receptor IL-21R on CD8^+^ T cells in liver and spleen, but the difference reached statistical significance for IL-21R on splenic CD8^+^ T cells only (*p* = 0.0216, Figures S3C,D in Supplementary Material). The majority of the increased IFN-γ^+^ CD8^+^ T cells were PD-1^+^ (*p* = 0.0079) (Figure 5H). Importantly, the absolute number of hepatic PD-1^+^IFN-γ^+^ CD8^+^ T cells positively correlated with liver MNC of CD25^−/−^CXCR3^−/−^ mice (Figure 5I). Of note, the average percentages of increased IFN-γ- or decreased IL-17A-producing CD4^+^ T cells resulted from deletion of CXCR3 that expressed PD-1 also increased, although the difference did not reach statistical significance (Figure S5B in Supplementary Material). Taken together, these results indicate that in CD25^−/−^ mice the inhibition of hepatic inflammation by CXCR3 is associated with reduced numbers of KLRG1^+^ T cells and PD-1^+^IFN-γ^+^ T cells in the liver.

### CXCR3 Deletion Decreases Levels of Pro-Inflammatory Factors in the Colon of CD25^−/−^ Mice

**Figure 6:**
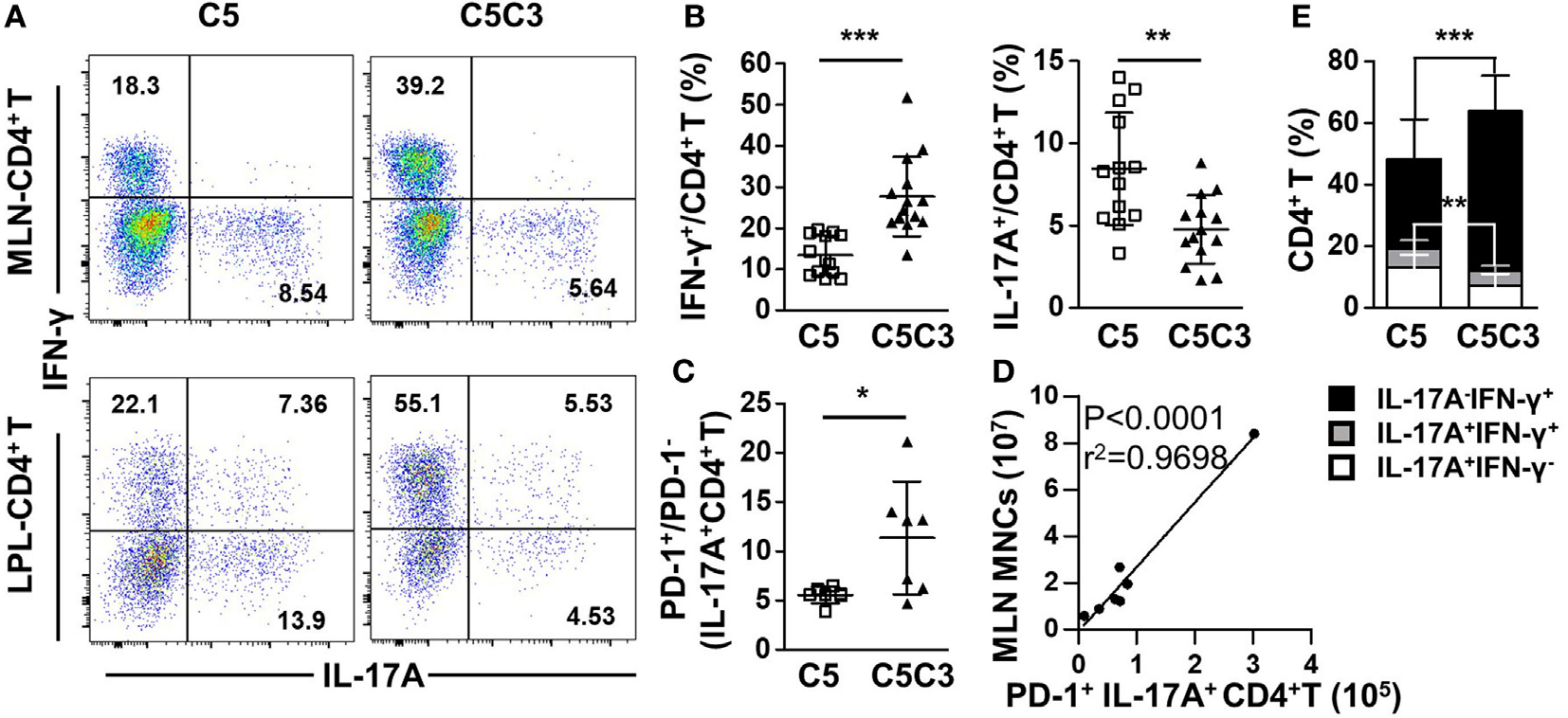
Effects of CXC chemokine receptor 3 (CXCR3) deletion on pro-inflammatory factors in colon of CD25^−/−^ mice. The phenotypes of T cell subsets in the CD25^−/−^ and CD25^−/−^CXCR3^−/−^ mice were analyzed with flow cytometry. **(A)** Representative flow cytometry dot plots of mesenteric lymph node (MLN) and colon lamina propria lymphocytes (LPL) CD4^+^ T cells stained for intracellular interferon (IFN)-γ and interleukin (IL)-17A. **(B)** Frequency of IFN-γ^+^ and IL-17A^+^ CD4^+^ T cells in the MLN from CD25^−/−^ mice (*N* = 13) and CD25^−/−^CXCR3^−/−^ mice (*N* = 14). **(C)** PD-1^+^/PD-1^−^ ratio in IL-17A-producing CD4^+^ T cells in the MLN, compared between CD25^−/−^ mice (*N* = 6) and CD25^−/−^ CXCR3^−/−^ mice (*N* = 7). **(D)** The correlation analysis between MLN mononuclear cells (MNCs) and colonic PD-1^+^IFN-γ^+^CD4^+^ T cells from CD25^−/−^CXCR3^−/−^ mice (*N* = 7). **(E)** Percentages of colon LPL CD4^+^ T cells expressing IFN-γ^+^ and/or IL-17A^+^, compared between CD25^−/−^ mice (*N* = 13) and CD25^−/−^CXCR3^−/−^ mice (*N* = 9). *p < 0.05; **p < 0.01; ***p < 0.001. C5, CD25^−/−^ mice; C5C3, CD25^−/−^CXCR3^−/−^ mice.

Deletion of CXCR3 in CD25^−/−^ mice resulted in increased frequency of IFN-γ-producing (*p* < 0.0001) and decreased frequency of IL-17A-producing (*p* = 0.0021) CD4^+^ T cells in MLN (Figures 6A,B), suggesting enhanced differentiation into Th1 instead of type 17 T help cell (Th17) cells caused by CXCR3 deletion. Although the percentages of CD4^+^ Tem and PD-1 on colonic CD4^+^ T cells were maintained (Figure S5C in Supplementary Material), the decreased IL-17A-producing CD4^+^ T cells were dominantly PD-1^+^ (*p* = 0.0299 for IL-17A^+^ CD4^+^ T) (Figure 6C). Importantly, the absolute number of MLN PD-1^+^ IL-17A^+^ CD4^+^ T cells positively correlated with MLN MNC of CD25^−/−^CXCR3^−/−^ mice (Figure 6D). Similar to the MLN, in the colonic LPL IFN-γ secreting ability of CD4^+^ T cells increased (*p* = 0.0005) and IL-17A secreting ability of CD4^+^ T cells decreased (*p* = 0.0019) as well after CXCR3 deletion in CD25^−/−^ mice (Figures 6A,E). However, the increased IFN-γ-producing CD4^+^ T cells were not dominantly PD-1^+^ (Figure S5D in Supplementary Material). By contrast, significant change was not observed in the percentage of IFN-γ^+^ CD8^+^ T cells in MLN and LPL in the same mice (Figures S5C,E in Supplementary Material). And the RNA levels of transcription factors *T-bet* and *RORγt* on MLN CD4^+^ T cells were same between CD25^−/−^ and CD25^−/−^CXCR3^−/−^ mice (Figures S3E,F in Supplementary Material). Taken together, these results indicate that in CD25^−/−^ mice the enhanced colonic inflammation by CXCR3 is associated with increased IL-17A^+^CD4^+^ T cells that dominantly express PD-1.

## Discussion

In this study, by using the CD25^−/−^ mouse model for both cholangitis and colitis we found increased expression of CXCR3 on T cells and increased levels of the CXCR3 ligands in the liver and colon in comparison to the controls. Deletion of CXCR3 from the CD25^−/−^ mice resulted in aggravated hepatic inflammatory responses with increased CD8^+^ T cells associated with their increased production of inflammatory cytokine IFN-γ in the liver, but alleviated colitis with decreased CD4^+^ T cells associated with their reduced production of IL-17A in the colon. These results are in agreement with our previous findings on the different roles of IFN-γ and IL-17A in regulating the severity of cholangitis versus colitis (10, 20). Together these findings indicate that the effects of CXCR3 on the autoimmune diseases were organ specific.

It was not clear whether CXCR3 plays the same regulatory role to the autoimmune T cells in the liver versus colon. A major function of CXCR3 is for the chemotaxis of immune cells, especially T cells (23, 24). CXCR3 is induced to be upregulated quickly and promotes migration of T cells, in particular effector CD4^+^ and CD8^+^ T cells, into specific organs (25, 26). In the CD25^−/−^ mice, we detected high levels of CXCR3 expression on T cells from the liver and MLN as well as increased levels of CXCR3 ligands (Figure 1), indicating that the CXCR3 pathway is involved in regulating autoimmune cholangitis and colitis in this mouse model. These results are in agreement with findings in PBC patients as well as another mouse model of PBC and colitis (10, 14, 27). However, it was not clear whether the effects of CXCR3 in these two autoimmune diseases were mediated by its chemotaxis function, or by its other immune regulatory function. To address this issue, we deleted CXCR3 from the CD25^−/−^ mice, which resulted in aggravated cholangitis but alleviated colitis (Figures 3 and 4). Of note, only CXCR3 deletion had no effect on liver and colon inflammation (Figure S1 in Supplementary Material). We found that deletion of CXCR3 from the CD25^−/−^ mice was associated with increased number of cytotoxic CD8^+^ T cells, in particular the KLRG1^+^CD8^+^ T cells with the reported ability of killing bile ducts (28), in the liver (Figure 5B). These findings support the pathogenic role of CD8^+^ T cells in the cholangitis, but are not consistent with CXCR3-mediated homing of T cells to the liver. Although the number of total CD4^+^ T cells increased in the liver as well, only CD8^+^ Tem cells but not the CD4^+^ Tem cells increased after CXCR3 deletion (Figures 3D,F). In contrast to the liver, the numbers of both CD4^+^ and CD8^+^ T cells from MLN and LPL decreased after CXCR3 deletion (Figures 4D,E; Figure S5E in Supplementary Material). This is in agreement with findings in inflammatory bowel disease (IBD) patients (29). Taken together, our results suggest that the role of CXCR3 in cholangitis is not mediated by its chemotaxis function, whereas CXCR3-mediated T cell homing to the gut could be a factor for the colitis of CD25^−/−^ mice. This is in agreement with previous report that CXCR3 is upregulated in gut-derived T cells compared to liver-derived T cells and that CXCR3^+^CD8^+^ T cells tend to reside in the colon (30). Since organ-specific chemokine receptors other than CXCR3 have been reported (31–33), we also examined expression of other chemokine receptors including CCR4, CCR5, and CCR6 but detected no significant difference on protein levels in liver between the CD25^−/−^ and CD25^−/−^CXCR3^−/−^ mice (Figure S4 in Supplementary Material), suggesting that these receptors were not the major factors affecting the autoimmune diseases in this mouse model. Previous study has reported different pathogenic effector cells for cholangitis and colitis as CD4^+^ T cells responsible for colitis and CD8^+^ T cells responsible for cholangitis (34). In our current study, we further showed that CXCR3 regulated these diseases with organ-specific mechanisms.

PD-1 pathway is critical for downregulation of immune response and maintaining peripheral tolerance (35–37). In both PBC patients and IBD patients, PD-1 is highly expressed on inflammatory T cells (38–40). It has been reported that the differentiation state of T cells is linked to positive treatment outcomes of PD-1 pathway blockage and that only Tem in both CD4^+^ and CD8^+^ T cell populations respond to PD-1 pathway regulation (41). In both the liver and colon of CD25^−/−^ mice, approximately 90% of PD-1^+^ T cells were Tem (Figure S1 in Supplementary Material). However, our data showed different expression patterns of CXCR3 and PD-1 on the pathogenic T cell subsets for colitis and cholangitis, the colon CD4^+^ T cells and the hepatic CD8^+^ T cells, respectively. Only approximately 10% of hepatic CD8^+^ T cells expressed both CXCR3 and PD-1, whereas 30% of colonic CD4^+^ T cells were CXCR3^+^PD-1^+^ (Figures 2C,D). In particular, deletion of CXCR3 in CD25^−/−^ mice lead to opposite results in cholangitis and colitis and increased percentage of hepatic PD-1^+^ cells (Figures 3, 4 and 5C). In the liver of CD25^−/−^ CXCR3^−/−^ mice, only the frequency of CD8^+^ Tem, but not that of CD4^+^ Tem, increased (Figure 3F). By contrast, the frequency of colon CD4^+^ Tem and the frequency of colon CD4^+^ T cells expressing PD-1 remained unchanged (Figure S5C in Supplementary Material). We proposed that the different expression patterns of PD-1 and CXCR3 on colonic CD4^+^ T and hepatic CD8^+^ T cells could be a factor for the different outcomes, considering the diverse function of CXCR3 in different environment (42). For cholangitis, based on the expression patterns of PD-1 and CXCR3 on the different hepatic T cell subsets, we speculated that CXCR3 might regulate the balance between effector and memory CD8^+^ T cell populations. Absence of CXCR3 but high level of PD-1 expression might promote the differentiation of Tcm into Tem cells in the autoimmune cholangitis, which is different from the observations in LCMV infection (43, 44) and will need to be further examined in future studies. In colon, considering the low frequency of CD4^+^ Tcm cells in CD25^−/−^ mice and unchanged PD-1 expression on CD4^+^ T cells after CXCR3 deletion, we proposed that the reduced migration of PD-1^+^ CD4^+^ T cells into the colon is primarily responsible for alleviated colitis.

Our results provide new information for the function of CXCR3 in autoimmune diseases. We showed that the deficiency of CXCR3 led to opposite outcomes for the severity of autoimmune cholangitis and colitis. For cholangitis, CXCR3 is likely to not only directly regulate CD8^+^ T cells and their IFN-γ production to ameliorate liver disease but also regulate CD4^+^ T cells to affect the function of CD8^+^ T cells indirectly. We propose, based on our findings, that deletion of CXCR3 might contribute to the differentiation of Tcm to Tem cells, not the proliferation of T cells (Figures S3A,B in Supplementary Material), especially in CD8^+^ T cells which was the cause for the increased T cells in the liver of CD25^−/−^CXCR3^−/−^ mice, as only Tem but not Tcm cells can migrate into non-lymphoid organs (26). Though effector CD8^+^ T cells require IL-21 from CD4^+^ T cells (45), we detected no significant difference on RNA levels of IL-21 on CD4^+^ T and IL-21 receptor (IL-21R) on CD8^+^ T cells in liver and spleen of CD25^−/−^CXCR3^−/−^ mice compared with CD25^−/−^ mice (Figures S3C,D in Supplementary Material). For the alleviated colitis in the CD25^−/−^CXCR3^−/−^ mice, our findings were in agreement with previous report for CXCR3 being a colon-specific T cell homing receptor (30). Besides, decreased proliferation ability of MLN T cells was another reason for alleviated colitis (Figures S3A,B in Supplementary Material). In addition, CXCR3 deficiency mediated inhibition of CD4^+^ T cell differentiation into Th17 cells was likely to be another driver for the alleviated colitis, although the relative mean fluorescent intensity (RMFI) of transcription factors *T-bet* and *RORγt* in both hepatic and MLN CD4^+^ T cells had no significant difference (Figures S3E,F in Supplementary Material). Previous studies of CXCR3 are mainly focused on CD4^+^ T cell response in a single organ. Th1 and Th17 responses are both decreased after CXCR3 deletion in a lupus nephritis model (7). Herein, we found different changes of Th1 and Th17 responses after CXCR3 deletion in both cholangitis and colitis, in which they resulted in opposite changes to the disease severity. We have reported CD8^+^ T cells, particularly IFN-γ^+^ CD8^+^ T cells are pathogenic cells for cholangitis and CD4^+^ T cells for colitis. IL-17A deficiency aggravates cholangitis but ameliorates colitis (10, 19, 20). For the opposite disease severity, we speculate the differentiation of CD8^+^ T cells was the main cause for aggravated cholangitis with second decreased Th17 response, while the decreased Th17 response was important for alleviated colitis after CXCR3 deficiency in CD25^−/−^ mice (Figures 5 and 6; Figure S5D in Supplementary Material). But the mechanisms and the role of CXCR3 especially in liver need to be further elucidated.

In conclusion, we have taken advantage of the CD25^−/−^ mouse model of autoimmune diseases in two distinct organs to reveal new regulatory effects of the CXCR3 pathway that does not involve its chemotaxis function. We also showed that deletion of a single factor CXCR3 resulted in opposite outcomes to the autoimmune diseases in different organs. Although the detailed mechanisms need to be further elucidated in future studies, our findings strongly suggest that extra caution should be taken for immunotherapy of organ-specific autoimmune diseases, as therapeutic interventions aiming at a target such as CXCR3 for certain disease could result in unexpected adverse effects in an unrelated organ.

## Ethics Statement

This study was carried out in accordance with the recommendations of Guide for the Care and Use of Laboratory Animals, USTC Animal Care and Use Committee. The protocol was approved by the USTC Animal Care and Use Committee.

## Author Contributions

Q-ZL, W-TM, J-BY, Z-BZ, and KY performed the experiments. Q-ZL analyzed the data. QM helped to analyze the HE results. Z-XL, YY, and Q-ZL designed and provided the funding of the project. Q-ZL wrote the manuscript. LL, MG, and Z-XL revised the manuscript.

## Conflict of Interest Statement

The authors declare that the research was conducted in the absence of any commercial or financial relationships that could be construed as a potential conflict of interest.

## Abbreviations

CXCR3, CXC chemokine receptor 3; CXCL9, chemokine (C-X-C motif) ligand 9; CXCL10, chemokine (C-X-C motif) ligand 10; IFN, interferon; IL, interleukin; LPL, lamina propria lymphocytes; MNCs, mononuclear cells; MLN, mesenteric lymph node; PBC, primary biliary cholangitis; Tcm, central memory T cell; Tem, effector memory T cell; Tn, naïve T cell; Th1, type 1 T help cell; Th17, type 17 T help cell.
