# Supplementary material for: The CXC Chemokine Receptor 3 Inhibits Autoimmune Cholangitis via CD8+ T Cells but Promotes Colitis via CD4+ T Cells
Source: Front Immunol. 2018 May 17;9:1090. doi: 10.3389/fimmu.2018.01090 (PMC5966573; doi:10.3389/fimmu.2018.01090)
Supplement: Supplementary file 1 [file presentation_1.PDF]

Supplementary Material

The Chemokine Receptor CXCR3 Inhibits Autoimmune Cholangitis via CD8$^+$ T Cells but Promotes Colitis via CD4$^+$ T Cells

Qing-Zhi Liu[1,2], Wen-Tao Ma[1,3], Jing-Bo Yang[1], Zhi-Bin Zhao[1,2], Kai Yan[1,2], Yuan Yao[1,2], Liang Li[1,2], Qi Miao[4], M. Eric Gershwin[5] and Zhe-Xiong Lian[1,2*]

Correspondence: Zhe-Xiong Lian: zxlian@scut.edu.cn

Supplementary Figure legends

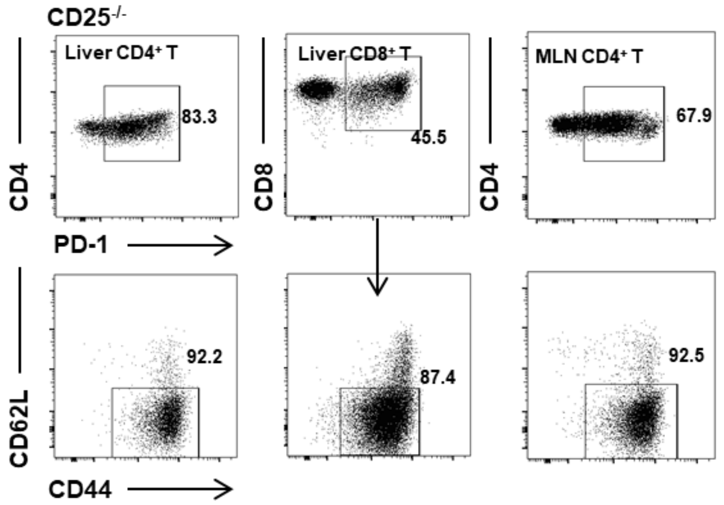

Supplementary Figure 1. PD-1^{+} T cells are mainly effector memory T cells of CD25$^{-/-}$ mice. Representative flow cytometry dot plots of PD-1, CD44and CD62L of CD4^{+} and CD8^{+} T cells in liver and MLN of CD25$^{-/-}$ mice.

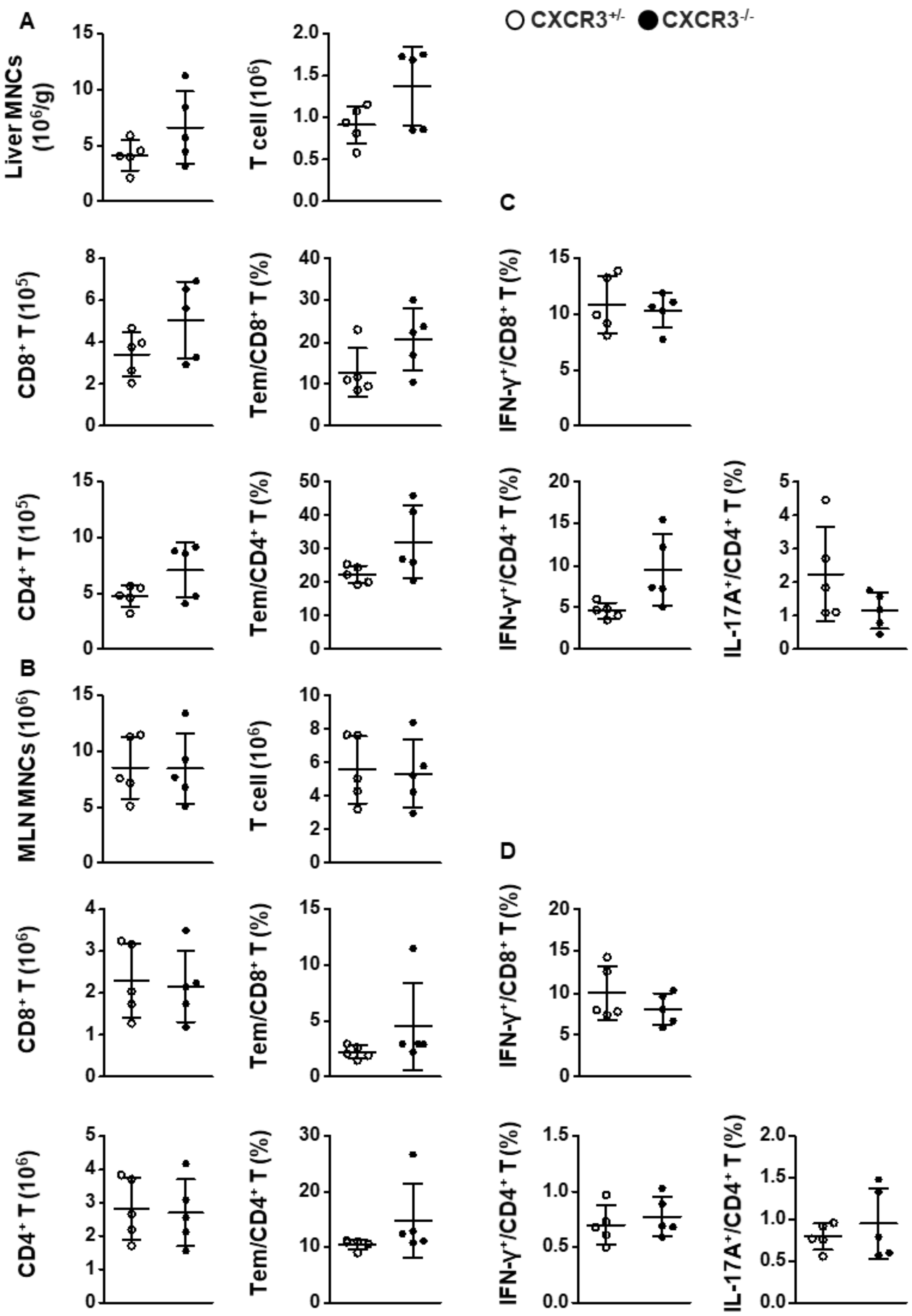

Supplementary Figure 2. CXCR3 deletion has no effects on T cells activation and function in liver and colon. Numbers of MNCs, T, $CD4^+$ T and $CD8^+$ T cells and percentages of effector memory (Tem) cells in $CD4^+$ T and $CD8^+$ T populations in liver (A) and MLN (B) of $CXCR3^{+/-}$ mice (N=5) and $CXCR3^{-/-}$ mice (N=5). Percentages of IFN-γ-producing $CD4^+$ T and $CD8^+$ T cells and IL-17A-producing $CD4^+$ T cells in liver (C) and MLN (D) of $CXCR3^{+/-}$ mice (N=5) and $CXCR3^{-/-}$ mice (N=5). * $P < 0.05$; ** $P < 0.01$; *** $P < 0.001$.

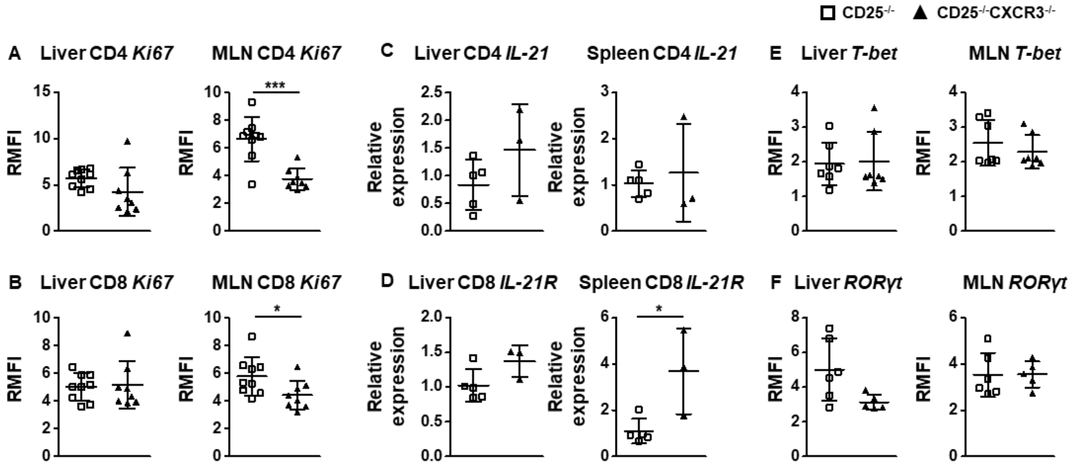

Supplementary Figure 3. CXCR3 deletion has on effects on the levels of transcription factors, proliferation and IL-21 of CD25$^{-/-}$ mice. Relative Mean Fluorescent Intensity (RMFI) of Ki67 on hepatic and MLN CD4^{+} T cells (A) and CD8^{+} T cells (B) in CD25$^{-/-}$ mice (N=9) and CD25$^{-/-}$ CXCR3$^{-/-}$ mice (N=8). The relative mRNA levels of IL-21 on CD4^{+} T cells and IL-21R on CD8^{+} T cells in liver (C) and spleen (D) were measured by quantitative real-time PCR and compared between CD25$^{+/-}$ mice (N=5) and CD25$^{-/-}$ mice (N=3). (E) Relative Mean Fluorescent Intensity (RMFI) of T-bet on hepatic and MLN CD4^{+} T cells in CD25$^{-/-}$ mice (N=7) and CD25$^{-/-}$ CXCR3$^{-/-}$ mice (N=7). (F) Relative Mean Fluorescent Intensity (RMFI) of RORγt on hepatic and MLN CD4^{+} T cells in CD25$^{-/-}$ mice (N=6) and CD25$^{-/-}$ CXCR3$^{-/-}$ mice (N=5). P <0.05; **P < 0.01; *** P < 0.001.

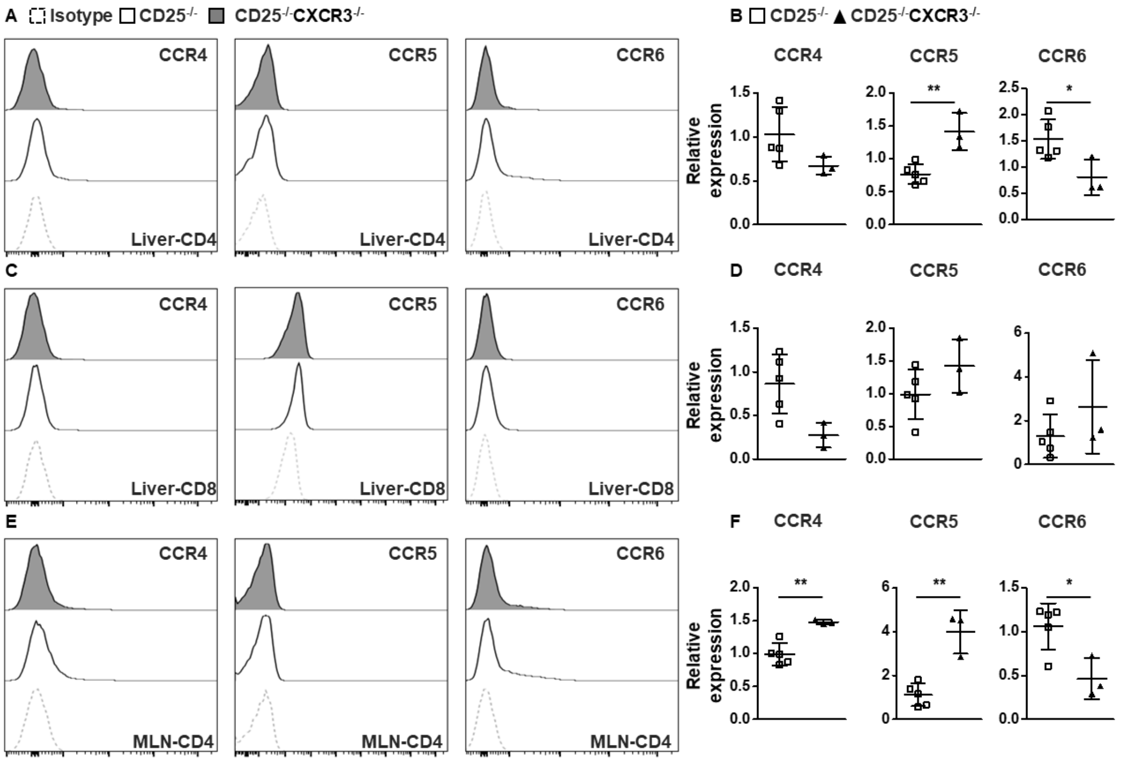

Supplementary Figure 4. Expressions of CCR4, CCR5 and CCR6 were unaffected by CXCR3 deletion of CD25$^{-/-}$ mice. Expression levels of CCR4, CCR5 and CCR6 on hepatic CD4^{+} T cells by flow cytometry (A) and RT-PCR (B) in CD25$^{-/-}$ mice (N=5) and CD25$^{-/-}$ CXCR3$^{-/-}$ mice (N=3). Expression levels of CCR4, CCR5 and CCR6 on hepatic CD8^{+} T cells by flow cytometry (C) and RT-PCR (D) in CD25$^{-/-}$ mice (N=5) and CD25$^{-/-}$ CXCR3$^{-/-}$ mice (N=3). Expression levels of CCR4, CCR5 and CCR6 on MLN CD4^{+} T cells by flow cytometry (E) and RT-PCR (F) in CD25$^{-/-}$ mice (N=5) and CD25$^{-/-}$ CXCR3$^{-/-}$ mice (N=3). P <0.05; **P < 0.01; *** P < 0.001

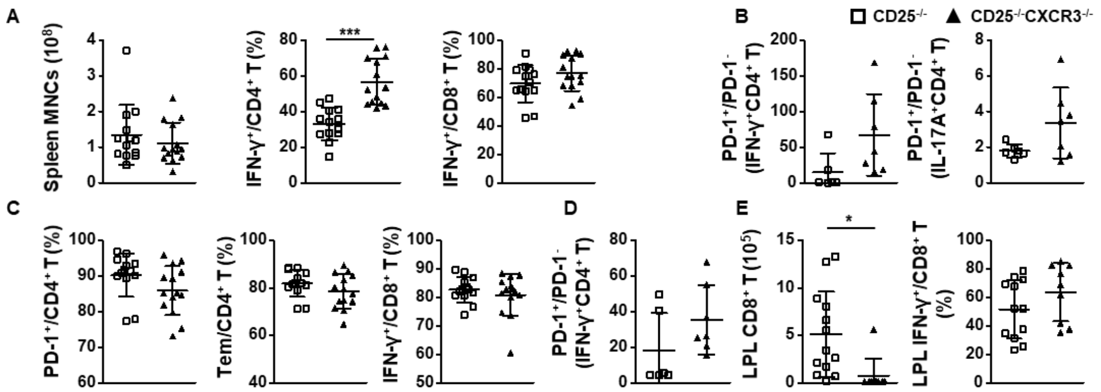

Supplementary Figure 5. CXCR3 deletion aggravates liver inflammation and alleviates colitis of CD25$^{-/-}$ mice. (A) Numbers of splenic MNCs and percentages of IFN-γ-producing splenic CD4^{+} T and CD8^{+} T cells in CD25$^{-/-}$ mice (N=13) and CD25$^{-/-}$ CXCR3$^{-/-}$ mice (N=14). (B) PD-1^{+} / PD-1^{-} ratio in IFN-γ- and IL-17A-producing hepatic CD4^{+} T cells respectively compared between CD25$^{-/-}$ mice (N=6) and CD25$^{-/-}$ CXCR3$^{-/-}$ mice (N=7). (C) Percentages of PD-1^{+} CD4^{+} T, CD4^{+} Tem and IFN-γ^{+} CD8^{+} T cells of MLN compared between CD25$^{-/-}$ mice (N=13) and CD25$^{-/-}$ CXCR3$^{-/-}$ mice (N=14). (D) PD-1^{+} / PD-1^{-} ratio in IFN-γ-producing CD4^{+} T cells in MLN of CD25$^{-/-}$ mice (N=6) and CD25$^{-/-}$CXCR3$^{-/-}$ mice (N=7). (E) Numbers of CD8^{+} T cells and percentage of IFN-γ^{+} CD8^{+} T cells in colon lamina propria lymphocytes (LPL) between CD25$^{-/-}$ mice (N=13) and CD25$^{-/-}$CXCR3$^{-/-}$ mice (N=9).

P <0.05; **P < 0.01; *** P < 0.001.
